# Implied Motion From Form Shows Motion Aids the Perception of Global Form in Amblyopia

**DOI:** 10.1167/iovs.61.5.58

**Published:** 2020-05-27

**Authors:** Mahesh R. Joshi, Anita J. Simmers, Seong T. Jeon

**Affiliations:** ^1^ Eye and Vision Research Group, School of Health Professions, University of Plymouth, Plymouth, United Kingdom; ^2^ Vision Sciences, Department of Life Sciences, Glasgow Caledonian University, Glasgow, United Kingdom

**Keywords:** motion perception, form perception, amblyopia, developmental disorder, dorsal stream vulnerability

## Abstract

**Purpose:**

Global motion and global form perception have been found to be abnormal in the presence of amblyopia. How such deficits manifest in visual function reliant on the interactions between these two visual processing mechanisms has not been adequately explored. In the current study, we use dynamic Glass patterns (dGlass) to measure implied motion thresholds in variable external noise to investigate the local and global limitations of processing.

**Methods:**

A total of 13 amblyopes (eight strabismic and five anisometropic, mean interocular visual acuity difference 0.30 ± 0.12 logMAR) and six visually normal controls discriminated the overall implied motion of dGlass generated by presenting nine independent sets of static Glass patterns over 0.5 seconds. The orientation of dipole elements was derived from the Gaussian distribution with prescribed mean and standard deviation that served as external noise. Thresholds at varying external noise were fitted to a set of linear amplifier models that were statistically compared to investigate the contribution of local and global processing parameters.

**Results:**

The implied motion thresholds were higher for strabismic (4.33° ± 1.34°) compared to anisometropic (2.32° ± 0.76°) amblyopia and controls (2.28° ± 0.50°) in the no-noise condition. The multivariate ANOVA analysis showed no difference between amblyopic and control observers at the no-noise and high-noise levels (*P* > 0.1). The statistical comparison of nested models showed normal internal noise and sampling efficiency parameters for both strabismic and anisometropic amblyopia (*P*S >0.50).

**Conclusions:**

The normal thresholds for implied motion in this study would suggest that motion aids the perception of global form cues present in dynamic Glass patterns. Our results challenge the proposed dorsal stream vulnerability in developmental disorders such as amblyopia.

According to the two-stream hypothesis,^1^^,^^2^ global motion and global form are thought to be processed independently within the dorsal and ventral streams, respectively.^3^^–^^6^ However, more recent studies suggest that motion and form processing might not be as independent as envisioned by the two-stream hypothesis.^7^^–^^10^ These studies report substantial interactions between motion and form processing mechanisms, with motion cues influencing perceived form and vice versa. For example, Ross et al.^11^ provided compelling evidence of how static form cues can induce motion perception using Glass patterns.

Glass patterns are created by superimposing a random dot pattern over an identical pattern after displacing the dot positions along a specified axis and separated by a finite displacement.^12^ The optimal displacement creates local orientation cues between the dot pairs (dipoles), and the perception of global orientation of the whole pattern arises when the orientations of dot pairs are consistent enough to be integrated to signal an overall orientation of the pattern.

When a set of static, independent Glass patterns with the same global orientation (such as diagonal translation) is displayed over time in a random sequence, the resulting perception is of induced motion (“implied motion”) along the orientation axis of the static Glass patterns. A display of static Glass patterns that induce a perception of reliable motion is known as a dynamic Glass pattern.^11^ In dynamic Glass patterns, unlike in real motion, the position of individual dots and dipole elements does not correspond from one frame to the next, eliminating reliable directional cues in motion vectors. Despite the absence of the sense of direction, a strong perception of motion is still perceived along the global orientation of the Glass patterns.

Imaging studies report that motion areas (middle temporal and middle superior temporal areas (MT/MST) complex) in humans^13^ and monkeys^7^ are equally stimulated by implied motion (using dynamic Glass patterns) and real directional motion (using random dot kinematograms). The activation of motion areas in response to dynamic Glass patterns is double to that of form processing areas such as V4.^13^ These findings suggest that despite the absence of directional cues, dynamic Glass patterns might be processed at least in part as a motion stimulus. How humans perceive motion from dynamic Glass patterns is still being investigated. One theory is that the dynamic Glass patterns stimulate a motion mechanism similar to that by motion streaks.^14^^–^^16^ The human visual system integrates temporal signals over a period of around 100 ms. During the window of time, local moving features are integrated to form “motion streaks” or “speedlines” along the direction of real motion.^15^ Independently varying the orientation of these motion streaks has a detrimental effect on the perception of the preceding motion direction.^14^ Burr and Ross^14^ suggested that the dipole pairs in dynamic Glass patterns could act as the endpoints of line segments forming motion streaks and hence provide motion cues based on the underlying structure of the Glass patterns.

Geisler^15^ proposed that outputs of both orientation and motion-selective cells in V1 are combined to form spatial motion direction sensors that are sensitive to both the direction of motion and orientation within the motion streak. More recent studies show that similar mechanisms might also be present at higher cortical processing areas such as MT and MST.^8^^,^^10^ Based on these findings, it has been proposed that the local processing (orientation of dipoles) of dynamic Glass patterns could occur by interaction at early cortical areas V1/V2,^13^^–^^18^ while motion and form interaction at extrastriate areas of MT, V4^8^^,^^10^^,^^13^^,^^19^^,^^20^ could be responsible for the global perception of implied motion.

Amblyopia, a neurologic disorder resulting in reduced vision in one or both eyes, is known to be associated with various global processing deficits in both motion and form perception.^21^^–^^26^ Notably, some researchers^27^^,^^28^ propose a dorsal stream vulnerability hypothesis in amblyopia and other developmental disorders where the processing of dorsal stream function (e.g., perception of global motion) is reported to be more compromised than ventral stream function (e.g., perception of global form). However, recent studies dispute this hypothesis on the ground that such differences in sensitivity could be stimulus specific rather than a general dysfunction of dorsal stream processing.^21^^,^^28^ In amblyopia, the site of visual processing deficits along both motion^21^^,^^25^^,^^26^^,^^29^ and form^21^^,^^23^^,^^24^ domains is reported to lie at extrastriate areas such as MT, MST, and V4, where local cues are integrated from early visual areas such as V1/V2, where no such deficits are found. However, it is not clear how such deficits manifest in tasks where both form and motion perception are intertwined as in implied motion perception. A few studies have investigated a similar question using alternative stimuli. For example, deficits in extracting structure from motion have been reported in amblyopia,^30^^,^^31^ whereas sensitivity to biological motion is reported to be mostly preserved.^32^ Previous experimental paradigms used to investigate the interaction between the two visual processing streams are not optimized to produce a systematic account for the difference, if any, in performance between normal and amblyopic observers when both form and motion processing are required for the task.

To assess global motion, form, and implied motion processing, researchers have measured the coherence threshold from the respective visual functions. In the coherence threshold paradigm, the minimum fraction of element dots/dipoles required to make a reliable judgment in the presence of random noise is determined, providing a combined measure of local and global processing mechanisms. Noise is a ubiquitous feature of all processing mechanism, including the human visual system.^33^ Noise can be decomposed into two main components: external noise, present in the stimuli, and internal noise—the noise within the visual system generated by sensory variances and decision uncertainties.^34^^,^^35^ Introducing varying levels of external noise to the task stimulus can disambiguate the local and global limitations of visual processing that can be described by a linear amplifier model (LAM).^34^^–^^36^ In such a paradigm, visual sensitivities are measured at variable external noise, and the change in performance can be modeled with the two parameters: internal equivalent noise (*σ_eq_*) and sampling efficiency parameters (*Eff*). In the LAM model, various sources of inherent limitations imposed by the visual system are collectively represented by a single component of internal noise (*σ_eq_*), and how the final perceptual decision is made is represented by the efficiency parameter (*Eff*).^34^^,^^35^

According to current literature, static Glass patterns are thought to be processed in two stages, the first where local orientation of the individual dipole pairs is processed in early visual areas such as V1, followed by the integration of these local orientation cues in higher extrastriate areas such as V4.^37^^–^^40^ For dynamic Glass patterns, the local processing of orientation would be similar to that of static Glass patterns while the overall implied motion is processed at higher extrastriate areas such as MT/MST.^20^ In line with this, the internal equivalent noise (*σ_eq_*) represents the uncertainty of the orientation processing mechanism at a local level (V1/V2), while the efficiency parameter represents the ability of the visual system to integrate local cues to provide the overall implied motion of the whole pattern, occurring at the extrastriate areas (MT, V4).^21^^,^^36^

In the current study, we designed an experiment using dynamic Glass patterns with a range of overall dipole orientation variances, which served as external noise to provide a systematic account of how different levels of processing (i.e., local versus global) affect the performance in form perception (orientation discrimination) when motion perception is induced at the same time in amblyopia.

## Methods

### Participants

A total of 13 amblyopes (eight strabismic and five anisometropic, mean ± SD interocular acuity difference [IOD] = 0.30 ± 0.12 logMAR and mean ± SD age = 28.30 ± 12.96 years) with IOD of ≥0.2 logMAR or history of amblyopia treatment (Table) and six normal controls (mean ± SD age = 28 ± 5.24 years) with normal or corrected-to-normal visual acuity were recruited. All experiments were conducted with the best (full) correction after refraction by an optometrist, who is one of the authors (MRJ). Informed written consent was obtained from each participant, and the study was carried out following the Code of Ethics of the World Medical Association, Declaration of Helsinki and approved by the Life Sciences Human Subjects Research Ethics Committee of Glasgow Caledonian University.

**Table.** Clinical Details of Amblyopic Participants

			Refraction			
Type	ID	IOD	OD	OS	Cover Test	Stereo (arcsec)
Strabismic	SS	0.41	+4.50/−0.50 × 172	+5.75/−1.00 × 22	Esotropia	No
	NJ	0.4	+1.00	+3.00	Esotropia	No
	CO	0.34	+4.00/−1.50 × 175	+4.50/−1.50 × 90	Intermittent esotropia	200
	HQ	0.50	−1.50/−2.00 × 5	−1.50/−2.00 × 5	Exotropia	No
	MR	0.26	+3.00/−2.50 × 90	+1.50	Esotropia	No
	JR	0.48	−2.50	−2.50	Esotropia	No
	KH	0.2	+8.50/−3.50 × 25	+9.00/−3.00 × 170	Esotropia	No
	JW	0.24	+0.75/−0.25 × 25	+3.25/−0.50 × 25	Esotropia	No
Anisometropic	RK	0.22	+1.75/−1.00 × 180	0.00	Exophoria	85
	KW	0.1	−6.25/−1.25 × 170	−6.50/−1.50 × 180	Exophoria	20
	HMc	0.26	−0.25	+1.00/−1.00 × 90	Exophoria	20
	MI	0.2	−3.50/−0.50 × 60	−8.50/−1.50 × 140	Exophoria	40
	KS	0.33	0.00	+2.00/−1.00 × 150	Exophoria	100

IOD, interocular difference, logMAR; OD, right eye; OS, left eye.

### Stimuli

The experimental stimuli were generated using MATLAB^41^ with Psychophysics Toolbox extensions^42^^,^^43^ and displayed on a 21-in. Sony Flatron monitor with a pixel resolution of 1920 × 1440 and refresh rate of 75 Hz powered by an Apple (Cupertino, CA, USA) computer with OS X. The dynamic Glass patterns were composed of nine frames of independently generated static Glass patterns displayed through 0.5-second stimulus duration in a circular aperture of 10° when viewed at 50 cm. Each static Glass pattern was generated by randomly placing 120 dots (of 0.166° diameter) around the center of the display; a copy of an identical set of dots was then superimposed at a displacement of 0.266° after linear geometrical transformation, creating a translation Glass pattern. The mean background luminance of the display and the element dot were 35 cd/m^2^ and 9 cd/m^2^, respectively, to achieve 95% Michelson contrast.

**Figure 1.**
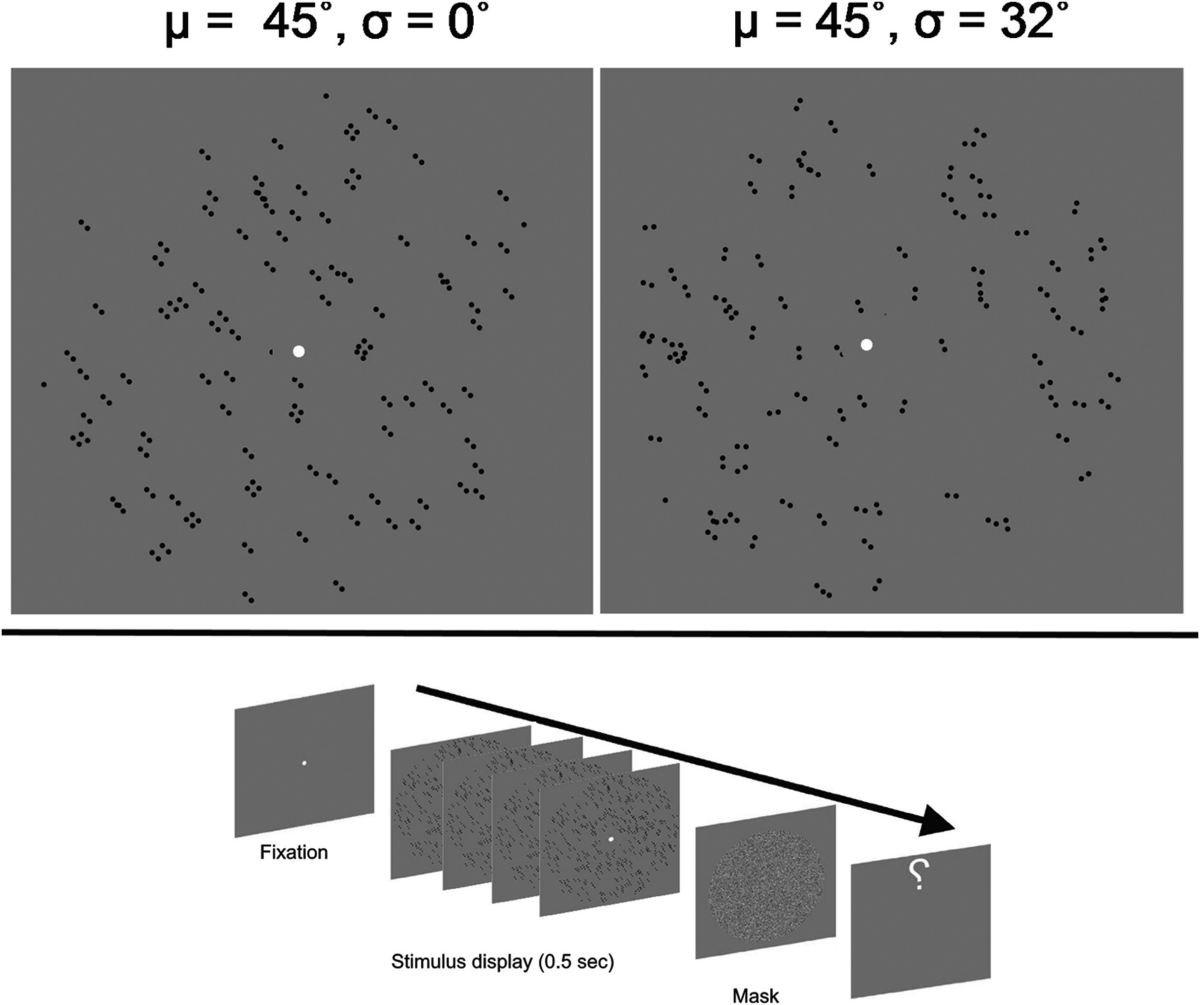
*Top panels*: Schematic representation of Glass patterns with differing orientation and noise. The orientations of individual dipoles are generated from a Gaussian distribution with the mean (µ) of the distribution representing the global orientation of the Glass patterns (45° from the vertical here). The increase in the standard deviation of the distribution (σ) increases the noise in the stimulus (*left*, low noise; *right*, high noise) with the global orientation remaining constant. The *bottom panel* shows a trial of dynamic Glass patterns display. The stimulus display consisted of nine independent static Glass patterns (four shown here) with the same global orientation presented within 0.50 seconds.

Each of the nine static Glass patterns was independently generated with the orientation of dipole elements derived from the Gaussian distribution of prescribed mean and standard deviation that served as external noise (Fig. 1). The increase in the standard deviation of the distribution increased the external noise with the mean of the distribution centered at different angles from the vertical reference (90°).

### Procedure

All participants completed the psychophysical experiment monocularly (amblyopic and fellow eyes for amblyopes, either dominant or nondominant eye randomly chosen for the normal controls) in a dark room with the computer monitor being the only source of light from 50 cm. At the start of the experiment, a white fixation dot of 0.2° diameter was presented at the center of screen, which was followed by the presentation of the dynamic Glass patterns for 0.5 seconds. A 10° diameter mask consisting of randomly generated pixel noise was then displayed for 0.25 seconds. The participant's task in each trial was to discriminate the overall implied motion of the dynamic Glass patterns from the reference of vertical (90°). Auditory feedback was provided for wrong responses.

A faster method of the equivalent noise paradigm^44^ was adapted for the data collection. Initially, the fine implied motion discrimination threshold was measured with no added external noise, and then the multiple (3×) of the offset obtained was used to evaluate the variance threshold targeting the high-noise condition. Both conditions were evaluated with interleaved three-down, one-up staircases. The staircase for the “no-noise” condition started with the mean implied motion of ±10° from the vertical while the staircase for the variance threshold was initiated with a preset implied motion threshold (3× no-noise threshold) as a mean offset from vertical with a standard deviation of 10°. Both staircases terminated after 10 reversals or 100 trials, whichever was reached first, and the thresholds were calculated as the geometrical mean of the last seven reversals.

### Modeling

The thresholds were modeled by the equation (1) to relate the performance to the added external noise (*σ_ext_*), internal equivalent noise (*σ_eq_*), and sampling efficiency (*Eff*) parameters.^34^^,^^35^

(1) $$\tau_{\mathrm{obs}}=\;\sqrt{\frac{\sigma_{\;\mathrm{eq}}^{2}+{\;\sigma }_{\;\mathrm{ext}}^{2}}{\mathrm{Eff}}}$$

Furthermore, the threshold data were used to fit various nested models from the full model defined by equation (1). The nested models were created from the full model by constraining different-fitting parameters (further details in the Results section). Among the nested models, nested *F*‐test (equation (2)) on the goodness of fit was used to statistically compare the models to determine whether any difference in threshold was best described by the change in either internal noise or sampling efficiency parameter.

(2) $$F\;\left(df_{1},df_{2}\right)=\;\frac{\left(r_{\mathrm{full}}^{2}-{\;r}_{\mathrm{reduced}}^{2}\right)/df_{1}}{\left(1-r_{\mathrm{full}}^{2}\right)/df_{2}}$$

where *d* *f*_1_ = *k_full_*  − *k_reduced_* and *d* *f*_2_ =  *N* − *k_full_.* *k* are the number of parameters in each model, and *N* is the number of predicted data points.

## Results

**Figure 2.**
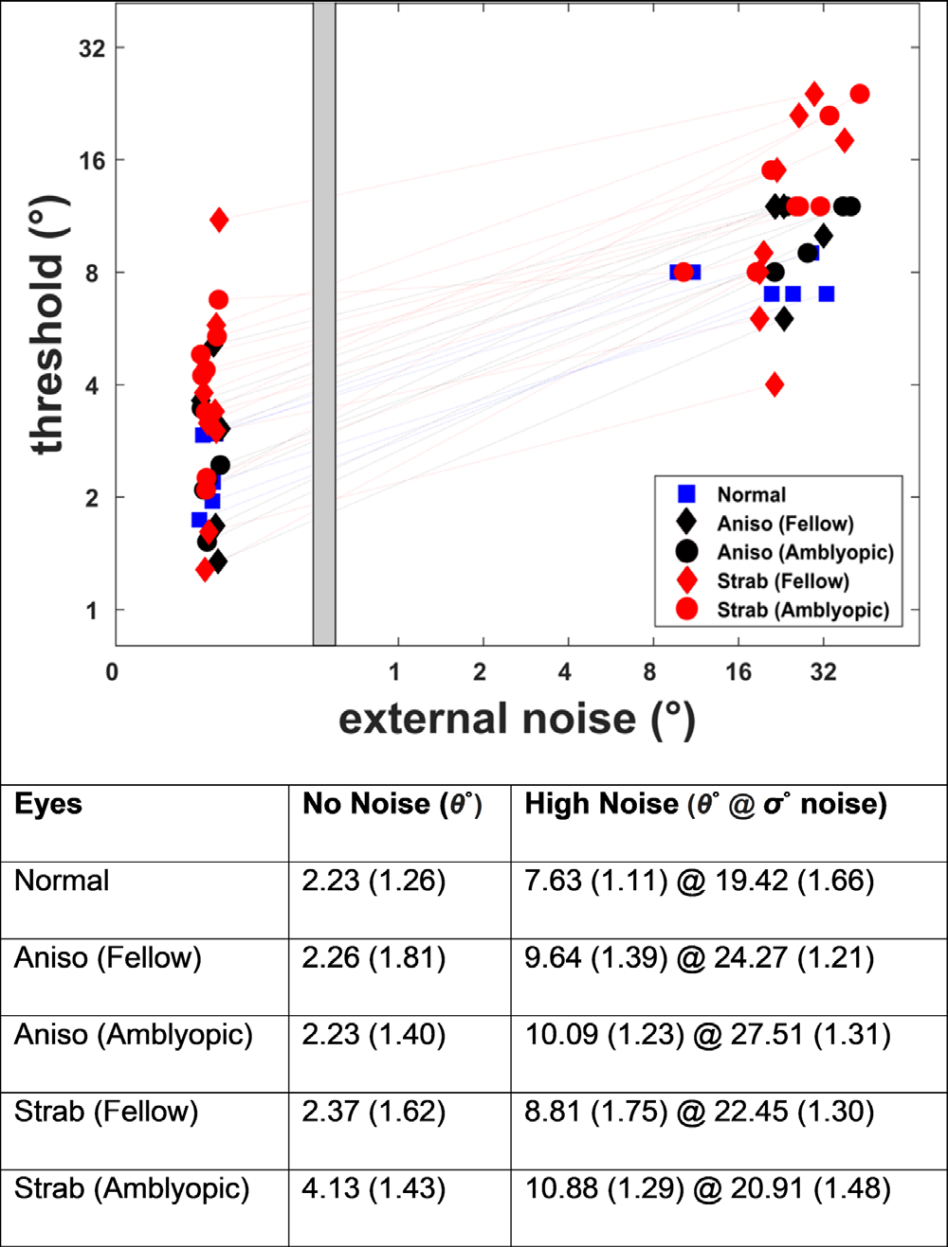
The scatterplot with individual implied motion thresholds at no-noise and high-noise levels for normal, fellow, and amblyopic eyes. The table provides mean thresholds at no-noise and high-noise conditions, and the values in the parentheses represent the standard deviation.

The mean implied motion thresholds for the amblyopic eye (2.32° ± 0.76°) and fellow eye (2.26° ± 1.34°) of anisometropic amblyopes were similar to that of the normal controls (2.28° ± 1.81°) for the no-noise condition. The mean threshold for the fellow eye (2.37° ± 1.62°) of the strabismic amblyopes was also similar to the normal thresholds. However, the threshold for the amblyopic eye of the strabismic amblyopes (4.33° ± 1.34°) was higher than that for normal at the low-noise condition (Fig. 2).

The log thresholds for noise levels and discrimination angles were analyzed using multivariate ANOVA. The log implied motion offset and noise levels (in orientation variance) were the two dependent variables, while the eye (five levels: strabismic amblyopic, strabismic fellow, anisometropic amblyopic, anisometropic fellow, and normal) was a fixed factor. Box's test of equality of covariance matrices showed that covariance of the dependent variable was similar between the *eye* (*M* = 22.24, *F* = 1.71, *P* > 0.05). The results showed no statistically significant difference in performance based on *eye, F*(8, 116) = 1.34, *P* > 0.1.

### Modeling

**Figure 3.**
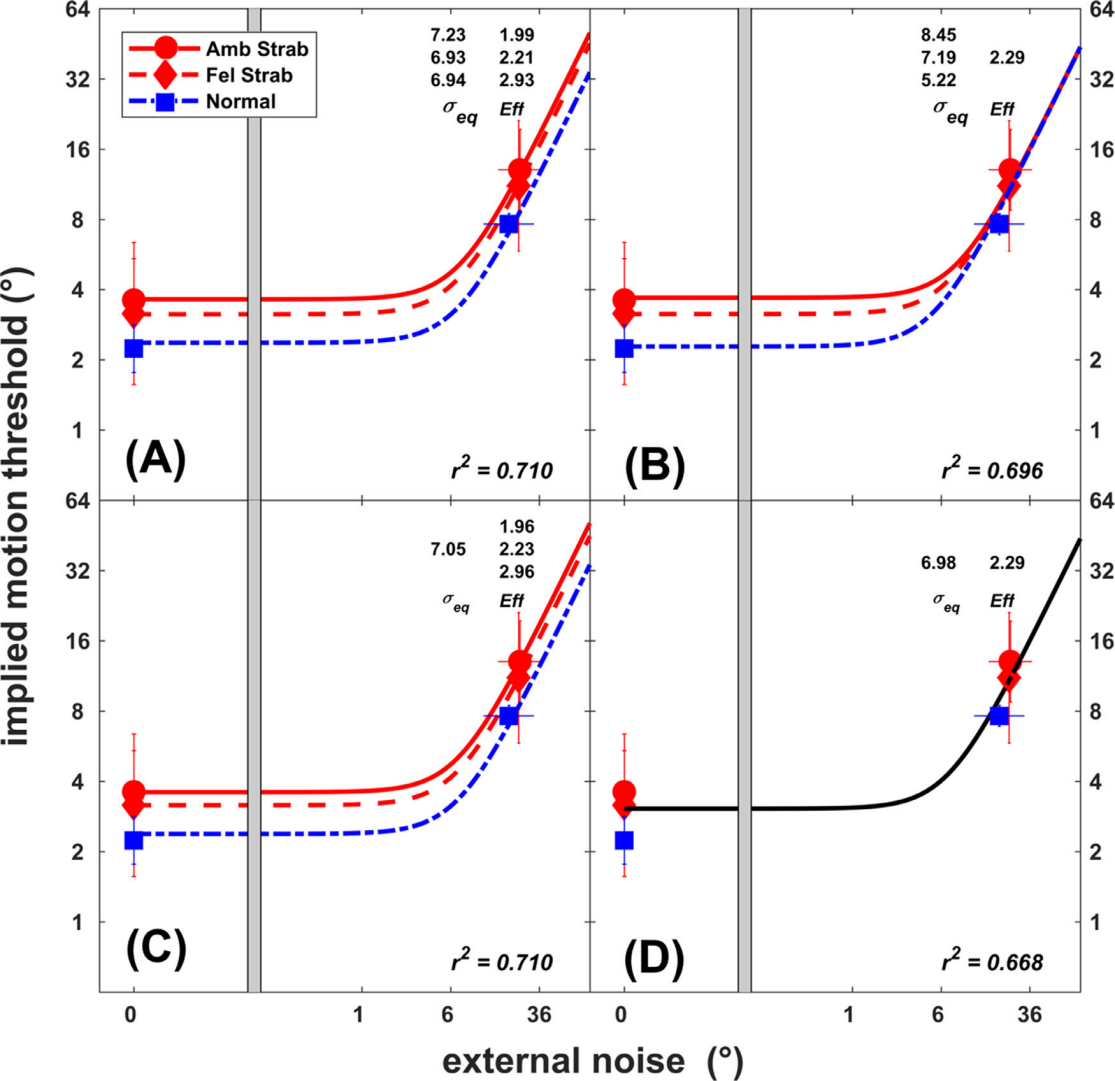
Nested models for the individual implied motion discrimination threshold data for dynamic Glass patterns in normal (*n* = 6), fellow (Fel Strab, *n* = 8), and amblyopic (Amb Strab, *n* = 8) eyes of strabismic participants, relating the implied motion offset thresholds and external noise to different values of the internal noise (*σ_eq_*) and sampling efficiency (*Eff*). The model parameters (*σ_eq_* and *Eff*) and goodness of fit (*r*^2^) of each model are also provided. The model with both parameters constrained across the amblyopic, fellow, and normal eyes (*bottom right panel*) was the best-fitting model to the threshold data. *Error bars* represent the standard deviation.

The implied motion thresholds were then fitted to the equivalent noise model (equation (1)) for strabismic participants. The full model (Fig. 3, A) consisted of six parameters, independent internal noise (*σ_eq_*), and sampling efficiency (*Eff*) parameters for each *eye* (normal, fellow eye of strabismic, and amblyopic eye of strabismic). The reduced models were created by restricting the fitting parameters (*σ_eq_*, *Eff*) across the eyes. The first restricted model (Fig. 3B) was fitted with four parameters. The efficiency (*Eff*) was constrained to take the same values for each eye group; hence, any difference in performance was represented by variation in the internal noise (*σ_eq_*) parameter. In the second model with four parameters (Fig. 3C), the internal noise (*σ_eq_*) was constrained, with variation in the efficiency (*Eff*) parameter representing any difference in performance. In the final model (Fig. 3D), both internal noise (*σ_eq_*) and efficiency parameters (*Eff*) were constrained to take the same values. The goodness-of-fit statistics for all restricted models were similar to the full model (full model, *r^2^*= 0.71; constrained models *r^2^* > 0.69): three *σ_eq_* and one *Eff* (*F*(2, 38) = 0.92, *P* = 0.41), one *σ_eq_* and three independent *Eff* (*F*(2, 38) = 0.001, *P* = 0.99), and the restricted model with one *σ_eq_* and one *Eff* (*F*(4, 38) = 1.37, *P* = 0.26).

The best model was hence chosen as the model with the least number of parameters (model with both *σ_eq_* and *Eff* constrained across the eyes), showing no difference in these parameters across the amblyopic and normal participants.

**Figure 4.**
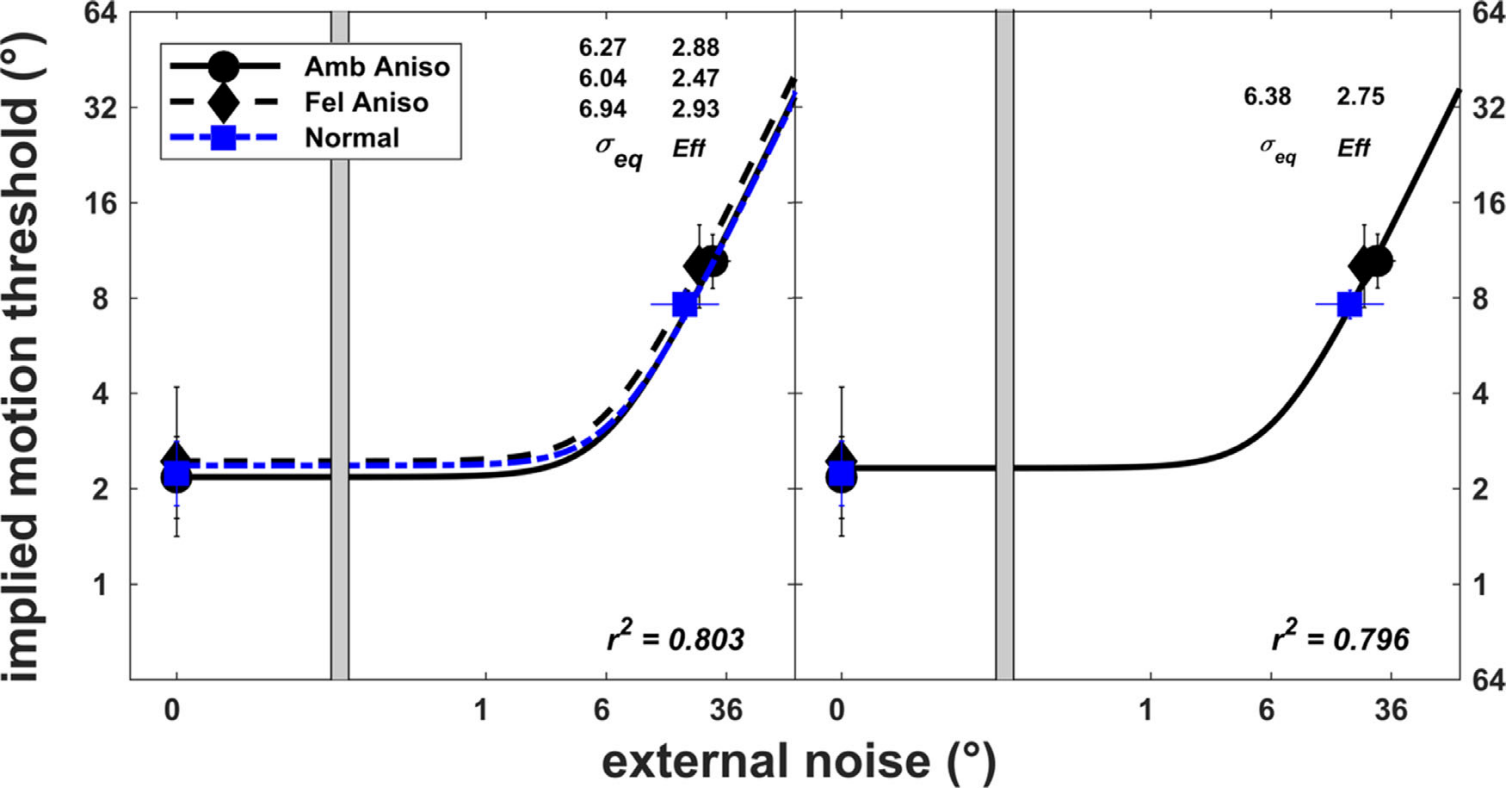
Nested models (*left*, full model; *right*, statistically best model) for the individual implied motion discrimination threshold data for dynamic Glass patterns in normal (*n* = 6), fellow (Fel Aniso, *n* = 5), and amblyopic (Amb Aniso, *n* = 5) eyes of anisometropic participants, relating the implied motion offset thresholds and external noise to different values of the internal noise (*σ_eq_*) and sampling efficiency (*Eff*). The model parameters (*σ_eq_* and *Eff*) and goodness of fit (*r*^2^) of each model are provided. *Error bars* represent the standard deviation.

The similar nested model analysis was also conducted for the anisometropic amblyopes (Fig. 4). The result showed that the most parsimonious model with both *σ_eq_* and *Eff* constrained across the normal, fellow eyes, and amblyopic eyes of anisometropic amblyopes best described the threshold data, *F*(4, 26) = 0.23, *P* = 0.92.

## Discussion

In this current study, the thresholds for implied motion in the dynamic Glass patterns were normal for amblyopic observers at both low- and high-noise conditions. The processing of dynamic Glass patterns relies on the inputs from both motion (dorsal stream) and form (ventral stream) processing mechanisms, but the extent of the contribution of each mechanism in the processing is unclear.^45^^,^^46^ Our results show that despite previously reported abnormal performance for both motion and form domains in amblyopia,^21^^,^^23^^–^^26^^,^^28^^,^^29^^,^^47^ implied motion thresholds that rely on the interaction between motion and form processing mechanisms appear normal.

As far as we know, this is the first study to evaluate the perception of implied motion using dynamic Glass patterns with an external noise paradigm in amblyopia. Other studies^30^^–^^32^^,^^48^ have evaluated the ability of amblyopes to integrate form and motion, with a structure from motion task. The perception of biological motion, in which the biological form is apparent only when the motion cues are present, is reported to be intact in amblyopes.^32^^,^^48^ Similarly, in a task where the orientation of a rectangle is apparent only when the constituent dots are in motion, the majority of amblyopes (11 of 12) performed normally at a speed of 5˚/s. However, the performance deteriorated at the slower dot speed of 0.1˚/s, demonstrating the importance of the stimulus parameters, such as speed, in probing a processing deficit.^30^ In the present study, the speed of the dynamic Glass patterns was relatively fast (18 Hz); hence, our normal findings are similar to those reported by Hayward et al.^30^ In another structure from motion task, however, amblyopes showed elevated thresholds for the detection of structure (depth) from motion using broadband dot stimuli as well as Gabor patches equated for the contrast deficit in the amblyopic eyes.^31^ The global amblyopic deficits for form (structure) from motion hence seem to be stimulus specific. Moreover, directly comparing our results using a motion-from-form task with studies based on the structure from motion tasks may not be representative as the visual processing mechanisms for both types of stimuli are not well understood and may well differ.

**Figure 5.**
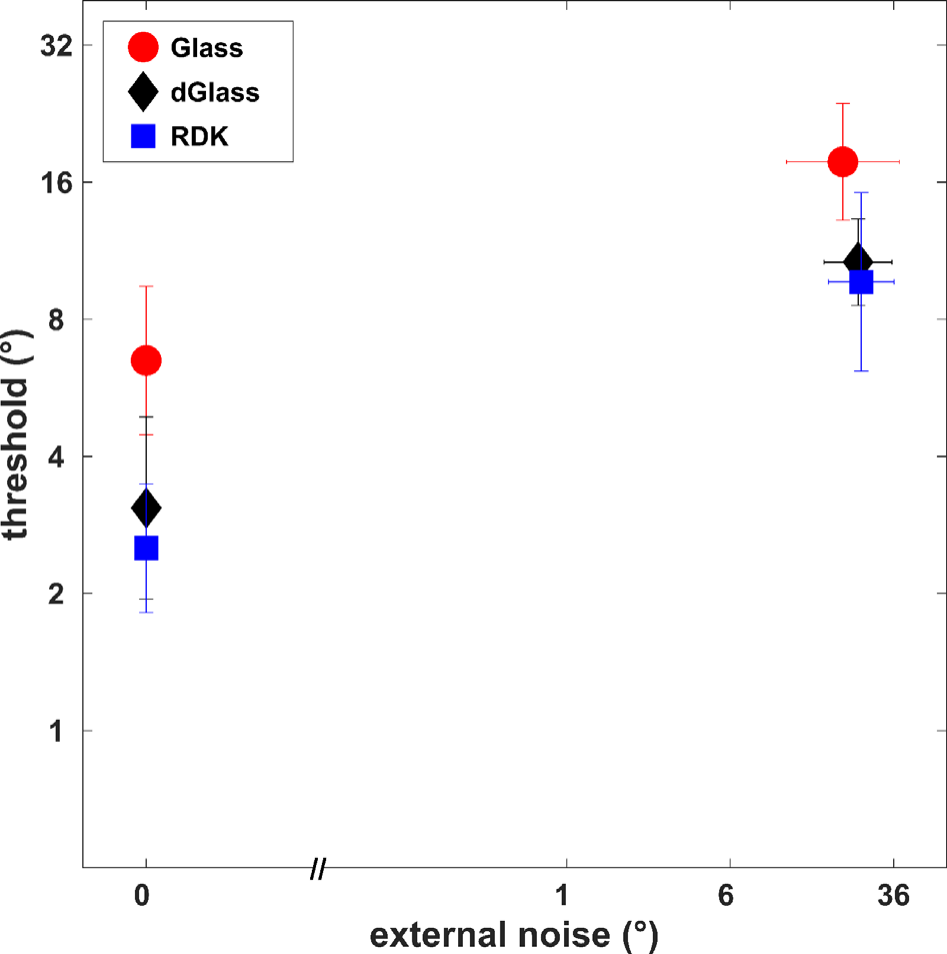
Mean thresholds (*n* = 11) for dynamic Glass patterns (dGlass) from amblyopic eyes (strabismic and anisometropic aggregated) in comparison to the static Glass patterns (Glass) and RDKs. *Error bars* represent the standard deviation.

Previous studies have shown that both global motion and global form are abnormal in amblyopia, with some studies suggesting more extensive deficits for motion processing.^23^^–^^26^^,^^47^ Employing similar stimulus parameters for both the motion and form domain as in the present study, deficits were also found in both motion and form domains but only for strabismic amblyopes.^21^ Figure 5 presents mean thresholds (*n* = 11, expect participants JR and MR) from the amblyopic eyes only (strabismic and anisometropic combined) for dynamic Glass patterns (dGlass) in comparison to the thresholds for static Glass patterns (stimulus display containing a single frame of Glass patterns [Glass]) and random dot kinematograms (RDKs; component dots exhibiting directional motion) reported in our previous study.^21^ The stimulus parameters such as dot and stimulus size, display duration, and contrast for static Glass patterns and RDKs were similar to the dynamic Glass patterns. The mean thresholds for dynamic Glass patterns were lower than the static Glass patterns but similar to the RDKs at both the no-noise and high-noise conditions (Fig. 5).

The nested modeling for dynamic Glass patterns revealed that both internal noise (*σ_eq_*) and sampling efficiency (*Eff*) parameters were normal in amblyopia. The internal noise parameter in such a paradigm relates to the local orientation uncertainty, while the efficiency relates to the global integration mechanism of the local cues.^21^^,^^35^^,^^36^ We report normal global and local processing of implied motion, despite previously reported abnormal global form thresholds for static Glass patterns.^21^ In addition, the thresholds for dynamic Glass patterns were similar to the RDKs and lower compared to the static Glass patterns (Fig. 5). These results suggest that the involvement of motion mechanisms in processing dynamic Glass patterns may aid the perception of global form in amblyopia. The mechanism behind how motion is perceived in dynamic Glass patterns despite the absence of directional motion cues is still being investigated. One of the theories suggests that the human visual system temporally integrates the dipoles from independent static Glass patterns, creating “motion streaks” that provide an impression of motion without direction.^11^^,^^14^^–^^16^ Other studies, however, suggest that the direct interaction between motion and form processing at V1/V2 and interaction between dedicated global motion areas (MT/MST) and global form area (V4) could be responsible for the local and global processing of dynamic Glass patterns, respectively.^8^^,^^10^^,^^20^ Moreover, these studies suggest a larger role for motion processing areas in decoding global implied motion from dynamic Glass patterns.^8^^,^^10^^,^^20^ The influence of motion processing areas in processing dynamic Glass patterns is further supported by physiologic and imaging studies in monkeys and humans that report similar activation of motion processing area MT+ by real motion in RDKs and implied motion in dynamic Glass patterns.^7^^,^^13^

Our results challenge the proposed dorsal stream vulnerability in developmental disorders such as amblyopia, where dorsal stream functions such as motion processing are reported to have a greater deficit than those of the ventral stream such as the processing of global form. The current results suggest that involvement of motion mechanisms may aid the perception of global form in amblyopia.
